# Follow-Up Visit Patterns in an Antiretroviral Therapy (ART) Programme in Zomba, Malawi

**DOI:** 10.1371/journal.pone.0101875

**Published:** 2014-07-17

**Authors:** Beth Rachlis, Donald C. Cole, Monique van Lettow, Michael Escobar, Adamson S. Muula, Farah Ahmad, James Orbinski, Adrienne K. Chan

**Affiliations:** 1 Dalla Lana School of Public Health, University of Toronto, Toronto, Canada; 2 Dignitas International, Zomba, Malawi; 3 College of Medicine, University of Malawi, Blantyre, Malawi; 4 School of Health Policy and Management, York University, Toronto, Canada; 5 Centre for International Governance Innovation, Waterloo, Canada; 6 Division of Infectious Diseases, Sunnybrook Health Sciences Centre, Toronto, Canada; University of Cape Town, South Africa

## Abstract

**Background:**

Identifying follow-up (FU) visit patterns, and exploring which factors influence them are likely to be useful in determining which patients on antiretroviral therapy (ART) may become Lost to Follow-Up (LTFU). Using an operation and implementation research approach, we sought 1) to describe the timing of FU visits amongst patients who have been on ART for shorter and longer periods of time; and 2) to determine the median time to late visits, and 3) to identify specific factors that may be associated with these patterns in Zomba, Malawi.

**Methods and Findings:**

Using routinely collected programme monitoring data from Zomba District, we performed descriptive analyses on all ART visits among patients who initiated ART between Jan. 1, 2007–June 30, 2010. Based on an expected FU date, each FU visit was classified as early (≥4 day before an expected FU date), on time (3 days before an expected FU date/up to 6 days after an expected FU date), or late (≥7 days after an expected FU date). In total, 7,815 patients with 76417 FU visits were included. Ninety-two percent of patients had ≥2 FU visits. At the majority of visits, patients were either on time or late. The median time to a first late visit among those with 2 or more visits was 216 days (IQR: 128–359). Various patient- and visit-level factors differed significantly across *Early*, *On Time*, and *Late* visit groups including ART adherence and frequency of, and type of side effects.

**Discussion:**

The majority of patients do not demonstrate consistent FU visit patterns. Individuals were generally on ART for at least 6 months before experiencing their first late visit. Our findings have implications for the development of effective interventions that meet patient needs when they present early and can reduce patient losses to follow-up when they are late. In particular, time-varying visit characteristics need further research.

## Introduction

With the number of people living with HIV receiving treatment with antiretroviral therapy (ART) increasing in recent years in sub-Saharan Africa [1], [2], patient retention remains an important challenge [3]–[8]. Disruption in care through missed scheduled visits can undermine both social (e.g., acceptance of a positive status [8] as well as clinical outcomes, including risk of virological failure) [9], [10]. While the discontinuation of ART can lead to drug resistance, HIV-related illnesses and death [11]–[17], individuals who miss visits in the first year of treatment have a higher mortality rate [18], [19]. A 2010 systematic review noted that by 2 years, ART programmes in sub-Saharan Africa retained approximately 70% of patients as high numbers of patients were lost to follow-up (LTFU) [3]. Determining relevant patterns of follow-up (FU) and exploring the factors associated with them can help to identify the patients that are at-risk of becoming LTFU, and when during the course of treatment, this risk is highest [4], [7], [8]. Such analyses can inform the development of evidence-based interventions that reduce attrition and improve patient outcomes.

Previous literature suggests that various risk factors have been associated with missing scheduled visits and becoming LTFU. Younger age at ART initiation [18], [20]–[24], type of ART regimen [25], location of ART management [21] and the occurrence of side effects [12], [26], [27] have been shown to be more important in the early stages of treatment. Risk of becoming LTFU and death has also been attributed to a poor clinical status at ART initiation indicated by: a low CD4 count [14], [17], [18], [27], a low body mass index [14], [26], initiation of ART at World Health Organization (WHO) clinical stage 3 or 4 [12], [20], [25], [28], and/or tuberculosis (TB) co-infection [23]. Later on, among patients who have been on ART for longer periods, feeling better and experiencing an improvement in health can lead to an increased risk of stopping ART and missing scheduled visits [12], as patients believe that treatment is no longer necessary. Other factors, including treatment literacy (e.g., understanding the natural course of treatment and the need to take medication as prescribed), also matter for treatment adherence [29], [30].

Malawi (adult HIV seroprevalence 10% [31]; population 15 million [32]) is one country in southern Africa that has achieved remarkable success in the scale-up of public access to ART. Between 2004 and September 2012, over 500,000 initiated ART [33]. According to the 2008 Malawian ART guidelines, patients who have missed a scheduled follow-up visit by more than two months and are not known to have to transferred out, died or stopped ART are considered LTFU and should be traced [34]. However, the high costs of tracing and a shortage of human resources necessary to find those who are missing has contributed to a backlog of LTFU patients with unknown outcomes [35], needing to be traced. Recent funding constraints and expanded initiation criteria [36] in Malawi have meant that existing ART resources must be stretched to meet the increased demand, making efficient use of the existing resources even more critical. While over 390,000 (73%) ART patients remain alive and on treatment in the national programme, over 90,000 (17%) have been LTFU [36].

We chose to take a strong operations and implementation research approach in the present study, considering a fuller spectrum of issues faced by front line healthcare workers. We wanted to work with real world conditions rather than attempt to control them [37]. Given that clinicians and front line healthcare workers face all types of patients and not only those who meet specific eligibility criteria, we sought to include a wide range of patients in order to explore the various follow-up visit patterns that can occur. More specifically, amongst patients who have been on ART for shorter and longer periods of time, we sought to 1) to comprehensively describe the time of FU visits, specifically identifying whether a patient on ART presents early, on time, or late for a scheduled follow-up visit; 2) to determine the median time to late FU visits and; 3) identify the specific patient and healthcare factors that are associated with different timing of FU visits in an ART programme in the Zomba District in southern Malawi.

## Materials and Methods

Dignitas International (DI), a Canadian non-governmental organization, has worked in partnership with the Malawi Ministry of Health (MOH) since 2004 to support delivery of comprehensive HIV care in the Zomba District, one of the most densely populated districts in Malawi (population: 670,500). District HIV prevalence is approximately 14.5% [37], although estimates within the district vary by location and population group [38]. Dignitas International supported the Malawi MOH to establish a tertiary referral HIV clinic at Zomba Central Hospital in 2004, and since 2006 has also supported the Zomba District Health Office to integrate HIV-related services into existing primary health services at 22 decentralized health centres throughout the district [21]. As per Malawian MOH guidelines, each patient who starts ART is given a unique treatment unit ART registration number. This number is written on a paper-based patient card called a Master Card and put into the electronic ART register for staff's use. All baseline registration data is entered at the time of ART initiation. Only ART follow-up visits were included in the present study as we were specifically looking at patterns among ART visits. At each ART FU visit, patient data is documented on the Master Card. Antiretroviral therapy is dispensed by an ART provider (e.g., clinical officer) or nurse. Under ideal conditions, patients initiated on ART are followed-up after two weeks and then are asked to return monthly. After 6 months, patients may be asked to return less often depending primarily upon provider assessments and drug availability [21], [34]. After the first 6 months, the current guidelines indicate that patients should be coming to the clinic every two or three months for dispensing and for a clinical assessment by the nurses. Patients may be flagged for a referral to a clinician at that time if needed. Data collection on MOH standardized registers and Master Cards are completed by clinicians and health staff at ART initiation and at each subsequent ART FU visit. Note, that unplanned visits may happen after which time ART dispensation may occur and as a result, the patient's pill count will be adjusted.

### Sample and Database

All adult patients who initiated ART and were followed-up between 1 January 2007 and 1 July 2010 were eligible for inclusion, with July 1 2010 being the last day for an expected FU visit. A total of 65 individuals initiated ART but did not have any further FU visits and were excluded in the present analyses. Children under the age of 15 were excluded. The data used in this study was extracted from routine monitoring and evaluation data gathered as part of the DI/Malawi MOH ART program in Zomba, Malawi, using standardized national Master Cards and registers. As all analyses were performed with de-identified data that was extracted from routine programmatic information, patients did not provide individual written or verbal consent to participate in the study. Ethical approval for data use was obtained from the University of Toronto HIV Research Ethics Board and the National Health Sciences Research Committee in Malawi.

### Overall Timing of Follow-up Visits

For each FU visit, a ‘days late’ value was determined. The expected return date was calculated by using the value for ART supply (in weeks) given at each FU visit. A patient's actual return date was compared to their expected return date. Note, that the 2008 ART guidelines recommended that the ART supply given at each FU visit includes an extra 2-day supply to act as a safety-buffer [34]. Visits in which patients arrived at the clinic between 3 days prior to and 6 days after an expected return date were classified as *On Time*. Patients often arrive at the clinic a few days prior to their scheduled return date for a variety of reasons including available transport. Hence those visits considered *Early* were those in which patients returned at least 4 days prior to an expected FU visit. Those classified as *Late* were those in which patients returned at least 7 days after an expected FU, consistent with the 7-days late value used to generate adherence proportions in the Malawi treatment guidelines [34]. As well, the clinical team in Zomba supported the use of *Late* (personal communication Gabriel Mateyu and Dr. Kevin Bezanson, Dignitas International, Zomba Malawi November 29, 2012).

Patients were first divided into those with 1 FU visit (generally, but not always, the two-week visit (87%)) and those ≥2 FU visits. Patients with 1 FU were categorized according to whether their visit was *Early*, *On Time*, or *Late*. Patients with ≥2 FU visits were categorized by a pattern of timing of their visits as: *Always Early*, *Always On Time*, *Always Late*, or *Other* (sometimes early, sometimes on time, sometimes late). Within the *Other* category, patients who were only *Early or On Time*, only *Early or Late*, and only *On Time or Late* were also reported. Among patients who had ≥2 FU visits, early visits were further stratified by whether a patient's visit was 4–7 days or ≥8 days early. Late visits were grouped by whether it was: 7–30 days, 31–59 days or ≥60 days late.

### Patient- and Visit-Level Characteristics Associated With Follow-Up Visit Patterns

**Figure 1:**
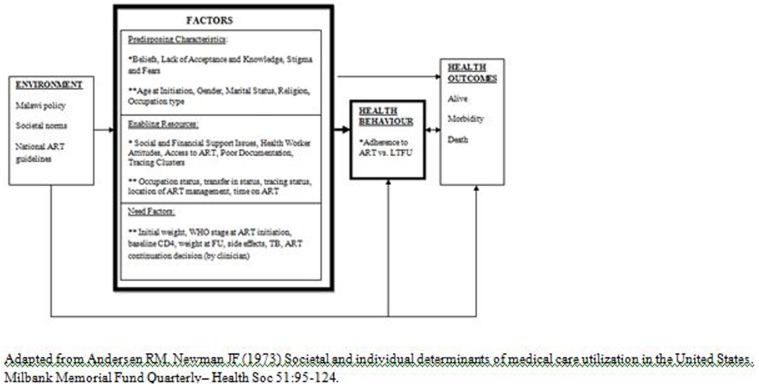
Conceptual model of potential determinants* of becoming LTFU from ART *bolded elements focused upon in this study.

Informed by the Andersen Newman Framework of Health Services Utilization [39], variables collected and explored were categorized as *predisposing characteristics*, *enabling resources* and *need factors* (Figure 1). According to the Andersen Newman Framework of Health Services Utilization [39], an individual's access to and use of health services is a function of three main types of factors: 1) *Predisposing Characteristics*, which are the socio-cultural characteristics of individuals that exist prior to their illness, 2) *Enabling Resources* which are the logistical aspects of obtaining care including personal, family, and community resources, and 3) *Need Factors*, which are the most immediate cause of health services use from problems that generate the need for care. For an individual living with HIV, such factors have the potential to affect not only their ability to return to the clinic on time for scheduled FU visits but also their adherence to ART.

#### Patient-Level Characteristics

The following variables collected at baseline were included. *Predisposing characteristics* included age at initiation (in years), gender (male vs. female), marital status (single, married, divorced/separated, widowed), and religion (Catholic, Protestant and non-Catholic Christian, Church of Christ/Church of God/Pentecostal, Seventh Day Adventist, Muslim). *Enabling Resources* included occupation status (working vs. not working), transferred into a DI-supported site at baseline (yes/no), whether the patient was ever traced (yes/no), and location of ART management (Centralized care: Zomba Central Hospital, Decentralized care: clinic with ≥35,000 outpatient visits/year, <35,000 outpatient visits per year). *Need factors* included mean weight at baseline (in kg) and WHO clinical stage at initiation (Stage 1, Stage 2, Stage 3, Stage 4).

#### Visit-Level Characteristics

At each follow-up visit, time on ART (in days) was reported as an *enabling resource*. *Need factors* included weight at FU (in kg), side effects from ART at FU (yes/no), ART continuation/whether there was a change in ART regimen at FU (no change, stopped, substituted a drug-for ART related drug toxicity from first line to alternative first line drug; switched regimen-due to toxicity, from a first line to a second line regimen, and held-due to drug toxicity with intent to restart again at some point in the future). Among patients who reported side effects, the number experiencing peripheral neuropathy (yes/no), rash (yes/no), or other side effects (yes/no) which included anemia, hepatitis, lactic acidosis, lipodystrophy, and pancreatitis were reported. Tuberculosis (TB) treatment outcomes (cured, failed, died, stopped, transferred out, unknown) were described when the patient had been treated for an episode of TB and an outcome was reported. Finally, ≥95% adherence to ART (yes/no) as determined by pill count was described. Note that in patients with 1 FU, side effects (yes/no), ≥95% adherence to ART (yes/no) and time on ART (in days) were reported only for that visit.

### Statistical Analysis

The analyses included all patients who had initiated ART between January 1 2007 and July 1 2010. We did not limit our analyses to only those patients with a specific duration of FU time (e.g., 1 year) to ensure that we could describe the various FU visit patterns that occur when patients have been on ART for a very short time in addition to a long time. For all continuous variables, the range, median and associated interquartile range (IQR) were assessed. Due to incompleteness of data, the number and percent missing were reported. When adequate normality warranted it, means and associated standard deviations (SD) were calculated. ANOVA or t-tests were used to explore statistically significant differences in continuous variables when appropriate. Proportions were described for all categorical variables. Chi-square tables were used to test for statistically significant differences in categorical variables between the different groups explored (see Patient-Level and Visit-Level Characteristics for grouping). Fisher's exact test was used when cell sizes in constructed chi-square tables were under 5. All analyses were conducted in STATA 12 (StataCorp, 2012)

#### Patient-Level Characteristics

Totals for each variable were reported for both the 1 FU and the ≥2 FU visit groups and differences in totals between groups were determined. Variables were also explored within visit time-groups: *Early* vs. *On Time* vs. *Late* among patients with only 1 FU visit; and *Always Early* or *On Time* vs. *Always Late* vs. *Other* among patients with ≥2 FU visits.

#### Visit-Level Characteristics

Multiple visits per patient were included (Median: 6, IQR: 3–11; Range: 1–39). Therefore, to account for the correlations between visits, generalized estimating equations (GEE) adjustments were utilized. Differences in visit characteristics among different timing of *Early* visits (4–7 days early vs. ≥8 days early) and or *Late* visits (7–30 days late vs. 31–59 days late vs. ≥60 days late) were explored. Totals for each variable were determined for each of the *Early*, *On Time*, and *Late* visits and differences across totals were also explored. In STATA 12 (StataCorp, 2012), the xtgee command was used with the cluster/group variable being the individual patient level. An exchangeable correlation structure was used given that we assumed that the correlation between visits of the same individual is assumed to be a constant (in the working covariance matrix). As our outcomes were binary (yes/no), we, essentially, were performing a logistic regression.

#### Median Time to First Late Follow-Up Visits

Stratified survival analyses were used to determine: a) the median time to a first FU late visit defined as above (i.e., ≥7 days late for a FU visit), b) median time to a first FU visit where a patient was ≥60 days and c) ≥90 days late. The number (and proportion) of patients and visits meeting these definitions were first described. Covariates explored include: gender (male vs. female), marital status (single vs. married), whether the patient had transferred into a DI-supported site (yes/no), type of care (decentralized vs. centralized) and WHO clinical stage at initiation (Stage 1 or 2 vs. Stage 3 or 4). For patients who had visits ≥60 and ≥90 days late, the number of visits where the patient had previously been ≥7 days late was reported. Analyses were first conducted for all patients and visits and then limited to those patients with ≥2 FU visits. Log-rank test was used to test for statistical differences across strata.

## Results

**Figure 2:**
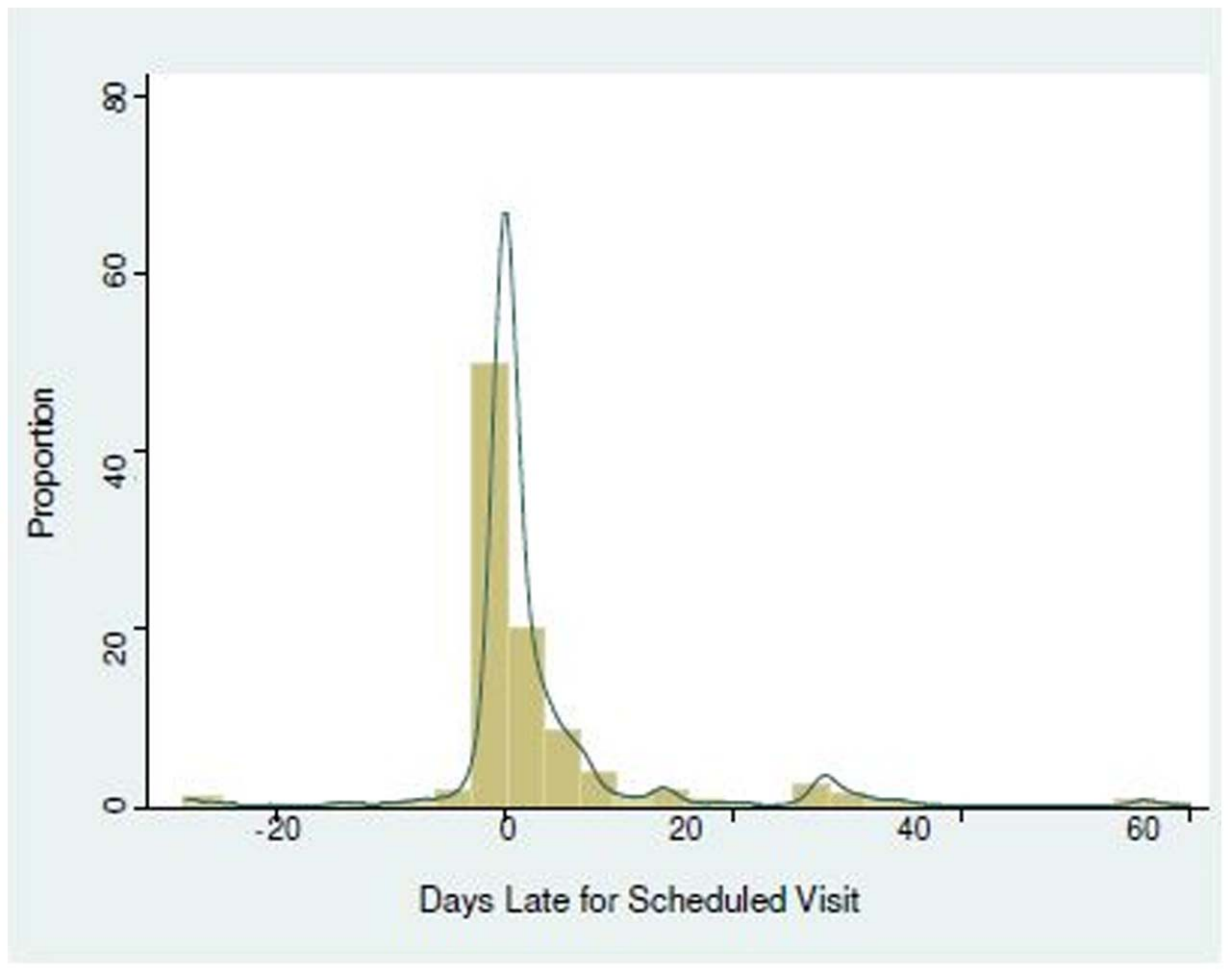
Patterns of follow-up among visits that were thirty days early up to 60 days late (n = 76,417).

**Figure 3:**
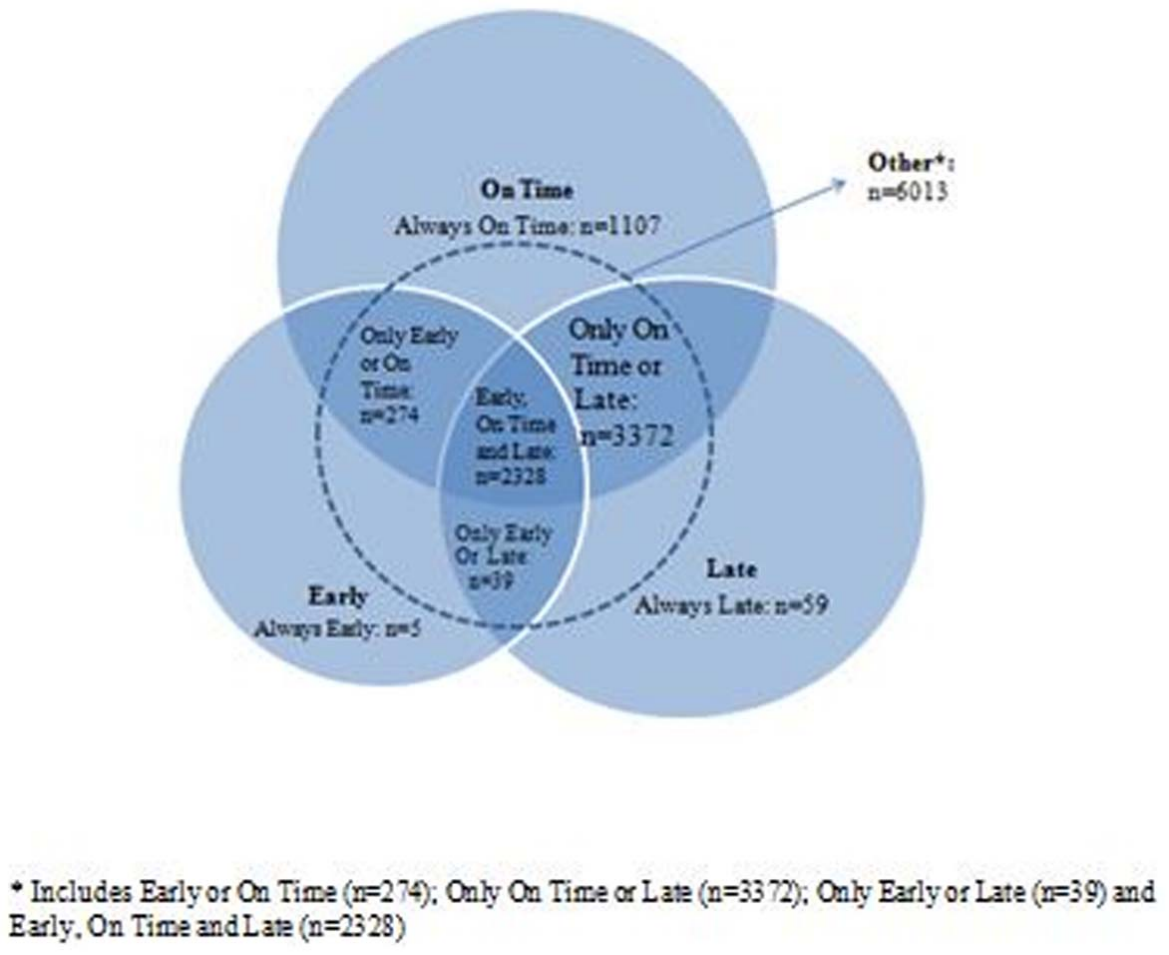
Patterns of follow-up for patients on ART with ≥2 follow-up visits (n = 7,184 patients).

In total, 7,815 patients with 76,417 FU visits were included (Table 1). In approximately 97% of visits, patients arrived at the clinic between 30 days prior to and 60 days after they were expected (Range: 83 days early up to 775 days late) (Figure 2). Six-hundred and thirty one patients (8%) had 1 FU visit after their initial baseline visit whereas 7,184 (92%) had ≥2 FU visits. Among patients with ≥2 FU visits (Figure 3), most visit profiles were a combination of being all of *Early, On Time* or *Late* at different visits (n = 6,013). Of these, the majority of visits were either *On Time* or *Late* (n = 3,372, 46.9%) or *Early, On Time* or *Late* (n = 2,328, 32.5%) depending on the visit. Fifteen percent (n = 1,107) of patients were *Always On Time*, five patients (<1%) were *Always Early*, and 59 (<1%) were *Always Late*. Among all patients, 75.7% (n = 5,914) had visits which were ≥7 days late, 23.4% (n = 1,830) had visits which were ≥60 days late and 12.3% (n = 967) of patients had visits which were ≥90 days late. Among patients with ≥2 FU visits, 80.4% (n = 5,773) of patients had visits which were ≥7 days late, 24.9% (n = 1,784) had ones which were ≥60 days late and 12.9% (n = 926) had ones which were ≥90 days late.

**Table 1:** Characteristics of all included patients (n = 7,815).

Variable	Summary
**Predisposing Factors**	
**Age (yrs)**	
Median (IQR)	35 (30–43)
Range	15–82
**Female** n (%)	5067 (64.7)
**Marital Status**	
Single	286 (4.2)
Married	3990 (58.3)
Divorced or Separated	1591 (23.3)
Widowed	944 (13.8)
**Religion**	
Catholic	1500 (21.5)
Protestant or Non-Catholic Christian	3034 (43.5)
Church of God or Pentecostal	1042 (15.0)
Seventh Day Adventist	279 (4.0)
Muslim	1022 (14.7)
**Enabling Factors**	
**Occupation Status**	
Working	7045 (97.6)
Not Working	171 (2.4)
**Transferred In (to Dignitas Supported Site)** n (%)	449 (5.8)
**Traced** n (%)	232 (3.0)
**ART Management Location**	
Centralized Care	3624 (46.4)
Decentralized Care >45,000 outpatient visits per year	1835 (23.5)
-Decentralized locations: <44,999 outpatient visits per year	2345 (30.0)
**Need Factors**	
**Weight at Baseline (kg)**	
Median (IQR)	50 (45–56)
Range	32–106.4
**WHO Stage at Initiation**	
Stage 1	261 (3.3)
Stage 2	3169 (40.6)
Stage 3	3401 (43.5)
Stage 4	983 (12.6)
	n = 1 presumed

Among the n = 1,830 patients who had visits which were ≥60 days late: 959 (52%) had no prior late visits; 457 (25%) were late once; 237 (13%) were late twice; 113 (6.2%) were late 3 times, and 65 (3.6%) had been late ≥4 times. Of n = 967 of all patients who had visits which were ≥90 days late: 536 (55.4%) had no prior late visits; 236 (24.4%) had been late once, 112 (11.6%) had been late twice, 55 (5.7%) had been late three times, 28 (2.9%) had been late ≥4 time.

Of n = 1,784 patients with ≥2 FU visits which had been ≥60 days late: 913 (51.1%) had no prior late visits; 456 (25.6%) had been late once; 237 (13.3%) had been late twice, 113 (6.3%) had been late three times and 62 (3.5%) had been late ≥4 times. Of n = 926 patients with at least 2 FU visits which were ≥90 days late: 498 (53.8%) had no prior late visits; 233 (25.2%) had been late once; 111 (12%) had been late twice; 55 (6%) had been late three times and 28 (3%) had been late ≥4 times.

### Patient-Level Characteristics

Overall, 64.7% of patients were women and the median age was 35 (IQR: 30–43). When compared to patients with ≥2 FU visits, a lower proportion of patients with only one FU visit were: women (58% vs. 65.3%, p<0.001), married (46% vs. 58.8%, p = 0.05), or receiving care at a decentralized ART management location (42.9% vs. 54.5%, p<0.001) (see Table 2). A higher proportion of 1 FU visit patients were non-Catholic Christian (47.2% vs. 38.3%, p = 0.008), had transferred into a DI-supported site at baseline (10.4% vs. 5.4%, p<0.001), or had ever been traced (4.4% vs. 2.8%, p = 0.02). At baseline, mean body weight was lower in the 1 FU visit group (50.3 kg vs. 51.5 kg, p = 0.001) and more patients initiated ART at WHO clinical Stage 4 compared to patients in the ≥2 FU visit group (16.8% vs. 12.2%, p<0.001). Patients with 1 FU visit had been on ART for a median of 45 days and 8.9% and 6.5% of patients reported non-adherence to ART and side effects, respectively.

**Table 2:** Patient Characteristics Associated with The Number and Type of Follow-up Visit Stratified According to The Anderson Newman Framework of Health Care Utilization (n = 7,815 patients).

	Only 1 follow-up visit (n = 631)					2 or more follow-up visits (n = 7,184)							
	Patient Groups					Patient Groups				Diff. between Groups			
	*Early n = 48*	*On Time n = 442*	*Late n = 141*	Diff. between groups	Total 1 FU	*Always Early n = 5*	*Always On Time n = 1,107*	*Always Late n = 59*	*Other n = 6,013*	Early/On Time vs. Late vs. Other	On Time vs. Late	Total ≥2 FU	DIFF. ACROSS TOTALS
**PREDISPOSING FACTORS**													
Age (yrs) at initation; Median (IQR), Range	35.5 (28–42, 20–78	35 (30–440, 15–79	33 (28–410, 15–76	p = 0.009	35 (29–440, 15–79	40 (28–40), 22–52	35 (30–43), 15–71	34 (27–42), 19–61	35 (30–43), 15–82	p = 0.613	p = 0.845	35 (30–43), 15–82	p = 0.347
Median duration on ART in days (IQR), Range	32 (17–35), 14–922	43 (42–740, 14–1176	100 (70–225), 41–1082	p = 0.001	45 (42–101), 14–1176								
% Female	64.6	56.8	59.6	p = 0.644	58	40	59.3	64.4	66.4	p<0.001	p = 0.983	65.3	p<0.001
Marital Status n (%) Single, Married, Divorced or Separated, Widowed	2 (5.0), 24 (60.0), 7 (17.5), 7 (17.5) n = 8 missing	18 (4.6), 211 (54.1), 106 (27.2), 47 (12.1) n = 60 missing	7 (5.6), 60 (48.0), 37 (29.6), 19 (15.2) n = 18 missing	p = 0.045	27 (4.3), 295 (46.0), 150 (23.8), 73 (11.6) n = 86 missing	0 (0), 3 (66.0), 0 (0), 0 (0) n = 2 missing	44 (4.6), 583 (60.4), 214 (22.2), 121 (12.5) n = 145 missing	1 (1.8), 31 (55.4), 14 (25.0), 8 (14.3) n = 5 missing	214 (4.1), 3078 (58.5), 1213 (23.1), 742 (14.1) n = 766 missing	p = 0.04	p = 0.100	259 (4.1), 3695 (58.8), 1441 (22.9), 871 (13.9), n = 918 missing	p = 0.05
Religion n (%) Catholic, Protestant or Non-Catholic Christian, Churct of God or Pentecostal, Seventh Day Adventist, Muslim	10 (21.3), 28 (59.6), 5 (10.6), 0 (0), 3 (6.4) n = 2 missing	94 (22.2), 187 (44.2), 73 (17.3), 12 (2.8), 49 (11.6) n = 27 missing	27 (20.6), 70 (53.4), 17 (13.0), 1 (<1.0), 15 (11.5) n = 11 missing	p = 0.503	131 (21.8), 285 (47.2), 95 (15.8), 13 (2.2), 67 (11.2) n = 40 missing	0 (0), 2 (67), 0 (0), 1 (33), n = 2 missing	198 (20.1), 434 (44.1), 151 (15.3), 39 (4.0), 144 (14.6) n = 141 missing	9 (16.1), 25 (44.6), 12 (21.4), 2 (3.6), 8 (14.3) n = 3 missing	1162 (21.8), 2288 (43.0), 784 (14.7), 224 (4.2), 803 (15.1) n = 652 missing	p = 0.540	p = 0.177	1369 (19.1), 2749 (38.3), 947 (13.2), 266 (3.7), 955 (13.3) n = 898 missing	p = 0.008
**ENABLING FACTORS**													
Occupation Status n (%) Working, Not Working	45 (95.7), 2 (4.3) n = 1 missing	438 (97.5), 11 (2.5) n = 3 missing	125 (94.0), 8 (6.0) n = 8 missing	p = 0.150	598 (96.6), 21 (3.4) n = 12 missing	3 (100) n = 2 missing	1003 (95.8), 23 (2.0) n = 51 missing	56 (94.9), 3 (5.1)	5385 (97.7), 124 (2.3) n = 504 missing	p = 0.171	p = 0.246	6447 (97.7), 150 (2.3) n = 587 missing	p = 0.08
Transferred In % Yes	4.2	11.4 n = 2 missing	9.3 n = 1 missing	p = 0.077	10.4 n = 3 missing	20	4.8	11.9	5.4	p = 0.045	p = 0.010	5.4	p<0.001
Traced % Yes	2.1	4.3	5.7	p = 0.336	4.4	0	3.3	3.4	2.7	p = 0.100	p = 0.153	2.8	p = 0.02
ART Management Location n (%)	n = 1 missing	n = 1 missing			n = 2 missing		n = 3 missing		n = 6 missing			n = 9 missing	
Centralized- Zomba Central Hospital	36 (76.6)	250 (56.7)	73 (51.8)		359 (57.1)	3 (60.0)	655 (59.3)	18 (30.5)	2589 (43.1)			3265 (45.5)	
Decentralized locations >45,000 outpatient visits per year	0 (0)	66 (15.0)	25 (17.7)	p = 0.015	91 (14.5)	1 (20.0)	249 (22.6)	19 (32.2)	1475 (24.6)	p<0.001	p<0.01	1744 (24.3)	p<0.001
Decentralized <44,999 outpatient visits per year	11 (23.4)	125 (28.3)	43 (30.5)		179 (28.5)	1 (20.0)	200 (18.1)	22 (37.3)	1943 (32.4)			2166 (30.2)	
**NEED FACTORS**													
Weight (kg) at baseline (exclude under 32 kg); Median (IQR), Range	49 (45–56), 33–69	49.8 (44–55), 32–104	50 (45–56), 33–80	p = 0.163	49.8 (44–55), 32–104	51 (48–55), 37–87	50 (45–57), 32–104	49 (44–55.2), 35–67	50 (45–56), 32–106.4	p = 0.107	p = 0.009	50 (45–56), 32–106.4	p = 0.001
WHO stage at initiation n (%) Stage 1, Stage 2, Stage 3, Stage 4	2 (4.2), 20 (41.7), 19 (39.6), 7 (14.6)	29 (6.6), 145 (32.8), 195 (44.1), 73 (16.5)	13 (9.2), 48 (34.0), 57 (40.4), 23 (16.3)	p = 0.110	44 (7.0), 213 (33.3), 271 (43.0), 103 (16.8)	1 (20.0), 0 (0), 3 (60.0), 1 (20.0)	78 (7.1), 428 (38.7), 448 (40.5), 152 (13.7) n = 1 presumed	4 (6.8), 22 (37.3), 26 (44.1), 7 (11.9)	134 (2.2), 2506 (41.7), 2653 (44.1), 720 (12.0)	p<0.001	p = 0.05	217 (3.0), 2956 (41.6), 3130 (43.0), 880 (12.2), n = 1 presumed	p<0.001
**BEHAVIOUR**													
Adherent, % No	13.2 n = 20 missing	7.1 n = 43 missing	13.5 n = 16 missing	p = 0.028	8.9 n = 72 missing								

Among patients with ≥2 FU visits, statistically different trends between patients. A lower proportion of females were *Always Early* (40%)/*Always On Time* (59.3%) versus *Always Late* (64.4%) versus the *Other* group (66.4%) (p<0.001). The proportion of patients who transferred into DI-supported sites was highest in the *Always Early* group (20%) and lowest in the *Always On Time* group (4.8%) although significant differences were noted in the *Early/On Time* vs. *Late* vs. *Other* (p = 0.045) and between the *On Time* (4.8%) vs. *Late* groups (11.9%) (p = 0.010). Statistical differences were noted between the *Early/On Time* vs. *Late* vs. *Other* group as well as the *On Time* vs. *Late* group for ART management (p<0.001 and p<0.01) and WHO clinical status at ART initiation (p<0.001 and p = 0.05). Significant differences in mean body weight at baseline were noted for *Always On Time* vs. *Always Late* (p = 0.009) (Table 2).

### Visit-level Characteristics

Among the 75,786 visits for those patients with ≥2 FU visits, 77% of visits (n  =  58,495) were *On Time*, 18% (n  =  13467) were *Late*, and 5% (n  =  3824) were *Early*. At a higher proportion of *Early* visits, patients reported side effects (19%) compared to *On Time* (11.4%) and/or *Late* (12.1%) visits (p<0.001) (see Table 3). A similar trend was observed for holding ART (p = 0.04). Among patients with *Early* visits, a higher proportion of patients reported side effects among visits which were ≥8 days early (20.8% vs. 13.9%, p<0.001). ART was more likely to be held at these visits (12.7% vs. 7.5%, p = 0.064) compared to visits where the visit was 4–7 days early although the difference was not significant in the latter. Mean body weight of patients was lower at ≥8 days early visits (54 vs. 55 kg, p = 0.014) and median time on ART (372 vs. 322 days, p = 0.002) was longer among patients who with 4–7 days early visits. Non-adherence to ART was highest among patients who came *Early* compared to those with *On Time* and/or *Late* visits (9.9% vs. 3.5% vs. 7.0%, p<0.001). A higher proportion of *Late* visits had patients reporting having a rash (53.4% vs. 38%) and ART was substituted more frequently (80.7% vs. 67.4%) particularly compared to *Early* visits. Although a lower proportion of patients reported side effects in visits in which they were ≥60 days late (9.5% vs. 12.4%), a higher proportion substituted their ART (85.3% vs. 78.7%) compared to when they were 7–30 days early. Patient visits ≥60 days late included patients who had been on ART for a longer median time compared to visits for *which* the patient was 7–30 days late (425 vs. 363 days) and mean body weight was lower (53 vs. 55 kg).

**Table 3:** Characteristics Associated with Early, On Time and Late Visits in Patients With ≥2 Follow-up Visits: (n = 75,786 visits among 7,184 patients).

	Early n = 3,824 visits among 2,604 patients				On Time n = 58,495 visits among 7,073 patients	Late, n = 13,467 visits among 5,773 patients					
	*8+ days n = 2,773*	*4–7 days n = 1,051*	Diff between columns	Total Early	Total On Time	*7–30 days: n = 8,820*	*31–59+ days: n = 2,613*	*60+ days n = 2,034*	Diff. between columns	Total Late	DIFF. BETWEEN TOTALS
Duration on ART (in days), Median (IQR) Range	372 (170–608) 7–1,234	322 (150–575), 21–1,199	**p = 0.002**	353 (162–600), 7–1,234	268 (128–492), 10–1,242	363 (191–608), 14–1,234	380 (223–626), 12–1,215	425 (267–669), 13–1,225	**p<0.001**	377 (211–620) 12–1,242	**p<0.001**
Weight at FU (in kg), Median (IQR), Range	54 (48–60), 32–104	55 (49–60), 35–98	**p = 0.014**	54 (48–60), 32–104	54 (49–60), 32–157	55 (49–60), 32–110	54 (48–60), 32–110	53 (48–59), 32–109	**p = 0.001**	54 (49–60), 32–110	p = 0.426
ART SE % Yes	n = 20.8%, n = 19 missing	13.9%, n = 3 missing	**p<0.001**	19.0%, n = 22 missing	11.4%, n = 267 missing	12.4%, n = 41 missing	13.2%, n = 16 missing	9.5%, n = 21 missing	**p = 0.02**	12.1%, n = 258 missing	**p<0.001**
SE Include:											
Rash, % Yes	38.6%	35.6%	p = 0.761	38.0%	40.3%	54.7%	49.7%	50.3%	p = 0.810	53.4%	**p<0.001**
Peripheral Neuropathy, % Yes	39.5%	39.0%	p = 0.966	39.4%	54.3%	45.6%	43.3%	40.3%	p = 0.914	44.3%	**p<0.001**
Other, % Yes	23.4%	26%	p = 0.701	23.8%	15.8%	18.4%	20.1%	28%	**p = 0.020**	19.9%	p = 0.328
ART continuation for those with SE, % n No Change, Stopped Substituted, Switched, Hold	97 (16.9), 12 (2.1), 381 (66.5), 9 (1.6), 73 (12.7) n = 5 missing	29 (19.9%), 3 (2.1%), 103 (70.6%), 0 (0%), 11 (7.5%)	p = 0.064	126 (17.6), 15 (2.1), 484 (67.4), 9 (1.3), 84 11.7) n = 5 missing	1770 (27.0), 19 (<1), 4643 (70.7), 52 (<1), 74 (1.1), n = 63 missing	205 (18.9), 3 (<1), 855 (78.7), 11 (1.0), 13 (1.2) n = 4 missing	43 (12.6), 2 (<1), 290 (84.8), 5 (1.5), 2 (<1)	21 (11.1), 0 (0), 162 (85.3), 2 (1.1), 5 (2.6)	**p = 0.001**	269 (16.6), 5 (<1),1307 (80.7), 18 (1.1), 20 (1.2) n = 4 missing	**p = 0.04**
TB treatment outcome where reported: Cured, Failed, Dead Stopped, Transfer Out	Out of n = 154 with reported outcome: 104 (67.5), 3 (1.9), 43 (27.9), 2 (1.3)	Out of n = 70: 48 (68.6), 0 (0), 4 (5.7), 18 (25.7)	p = 0.265	Out of n = 222: 152 (68.5), 0 (0), 7 (3.2), 61 (27.5), 2 (<1)	Out of n = 3101: 2272 (73.3), 6 (<1), 60 (3.4), 698 (22.5), 20 (<1)	Out of n = 485: 345 (71.1), 1 (<1), 13 (2.7), 122 (25.2), 4 (1)	Out of n = 139: 109 (78.4), 0 (0), 3 (2.2), 27 (19.4), 0 (0)	Of n = 81: 60 (74.1), 1 (1.2), 1 (1.2), 18 (22.2), 1 (1.2)	p = 0.164	Out of n = 705: 514 (72.9), 2 (<1), 17 (2.4), 167 (23.7), 5 (<1)	p = 0.344
Adherent, % No	7.8%, n = 520 missing	15.2% n = 140 missing	**p<0.001**	9.9%, n = 949 missing	3.5%, n = 2702 missing	7.4%, n = 532 missing	6.6%, n = 194 missing	6.8%, n = 216 missing	**p<0.001.**	7.0%, n = 952	**p<0.001**

*n.s. = not significant, ART = antiretroviral therapy, TB =  Tuberculosis, IQR = interquartile range, FU = follow-up; SE = side effects.

Patients at *On Time* visits had the shortest median time on ART (268 days) compared to both *Early* (353 days) and *Late* (425 days) visits (p<0.001). Although the proportion reporting side effects was lowest in *On Time* visits (11.4%), peripheral neuropathy was reported most frequently (54.3%).

### Median Time to First Late Follow-Up Visits

The median time to a first late visit among all patients and only those with ≥2 FU visits were 303 days (IQR: 183–497) and 216 days (IQR: 128–359) respectively. The median time to a first ≥60 days late visit in all patients and in those with ≥2 visits were 388.5 days (IQR: 245–650) and 393.5 days (251–651) respectively. The median time to a first ≥90 days late visit were 399.5 days (IQR: 274–633) and 406 days (IQR: 279.5–638) respectively. Individuals who were male, unmarried, in care at a decentralized site and whom initiated ART at WHO clinical stage 3/4 generally had a shorter time on ART before a first late visit (Table 4). Patients who transferred into a DI-supported site had a longer time to a first ≥7, ≥60 and ≥90 days late visit for all patients as well as those with ≥2 FU visits (p<0.001 in all comparisons).

**Table 4:** Median Time to a First Late Follow-Up Visits by ≥7 days, ≥60 days and ≥90 days Stratified By Baseline Predisposing Characteristics, Enabling Resources and Need Factors Among All Patients and Those with at Least 2 FU Visits.

	All patients (n = 7,815)				≥2 Follow-Up Visits (n = 7,184)			
	Median Time (IQR) to first ≥7 days late visit n = 5,924	Median time (IQR) to first ≥60 days late visit n = 1,830	Median time (IQR) to first ≥90 days late visit n = 967	Log-Rank	Median time (IQR) to first ≥7 days late visit n = 5,773	Median time (IQR) to first ≥60 days late visit n = 1,784	Median time (IQR) to first ≥90 days late visit n = 926	Log-Rank
**PREDISPOSING FACTORS**								
**Gender**								
Male	302 (182–482) n = 1982	356 (238–583) n = 656	383 (278–583) n = 302	p = 0.05 for ≥60 and ≥90 days late	210 (127–347) n = 1925	363 (141–592) n = 552	395 (282–609) n = 321	p = 0.04 for ≥60 days late; p = 0.05 for ≥90 days late
Female	305 (184–508) n = 3932	405 (252–670) n = 1265	406 (273–648) n = 665		219 (131–365) n = 3841	406 (256–677) n = 1232	412 (279–655) n = 605	
**Marital Status**								
Single	196 (116–329) n = 2146	351 (230–558) n = 732	370 (267–551) n = 396	p = 0.05 for ≥7 days late p = 0.04 for ≥60 days late at p = 0.03 for t≥90 days late	196 (122–330) n = 2083	356 (235–561) n = 711	389.5 (267–609) n = 378	p = 0.09 for ≥7 days late p = 0.045 for ≥60 days late at p = 0.02 for ≥90 days late
Married	211 (126–351) n = 3778	385 (247–636) n = 1098	390 (385–605) n = 571		214 (128–353) n = 2963	388 (252–645) n = 1073	415.5 (295–655) n = 548	
**ENABLING FACTORS**								
**Transferred In**								
Yes	398 (230–639) n = 330	664 (444–846) n = 95	701 (466–815) n = 46	p<0.001 for all	397 (213–637) n = 309	700 (441–631) n = 88	701 (434–830) n = 43	p<0.001 for all
No	210 (126–342) n = 5556	374 (243–622)n = 1725	386 (269–608) n = 921		210 (122–345) n = 5437	382 (245–631) n = 1696	390 (267–614) n = 883	
**Type of Care**								
Centralized	212 (113–362) n = 2511	439 (270–709) n = 455	449 (300–7220 n = 191	p<0.001 for ≥60 and ≥90 days late	218 (123–365) n = 2436	446 (280–722) n = 438	472 (314–749) n = 179	p = 0.03 for ≥7days late, p<0.001 for ≥90 days late
Decentralized	212 (133–356) n = 3403	373 (242–622) n = 1437	385 (273–603) n = 776		212 (133–365) n = 3337	377 (243–629) n = 1436	391 (274–660) n = 746	
**NEED FACTORS**								
**WHO Stage**								
Stage 1 or 2	240 (139–404) n = 2566	424 (273–736) n = 762	444 (286–686) n = 391	p<0.001 for all	247 (138–410) n = 2528	444 (273–542) n = 739	455 (289–691) n = 394	p<0.001 for all
Stage 3 or 4	191 (125–318) n = 3358	334 (219–536) n = 1068	350 (252–519) n = 576		196 (124–321) n = 3245	338 (226–542) n = 1045	359 (254–522) n = 552	

## Discussion

To our knowledge, this is the first study that comprehensively described the various patterns of follow-up visits and their timing, specifically stratifying visits as early, on time or late among patients who had been on ART for different periods of time. In the present study, we found that individuals in our setting were generally on ART for over 6 months before experiencing their first late visit, although this varied across patients. Furthermore, while a proportion of patients in this study were *Always Early, Always On Time*, or *Always Late* for their follow-up visits, the majority of patients did not demonstrate a consistent pattern across their visits. Our decision to include patients with a range of FU times (versus only including patients with minimum duration of FU time, e.g., 1 year), was based on our desire to understand the patterns of FU visits that can occur in the early as well as the later stages of treatment. Clinicians and front line healthcare workers deal with all types of patients including those who have just recently initiated ART. By taking a strong operations and implementation research approach [37], we sought to shed light on the various patterns of FU visits that can occur among patients who have been on treatment for shorter versus longer periods of time. As well, by not setting a minimum duration of FU time, we were able to explore patients who are at risk of becoming LTFU in the very early stages of treatment. Understanding why some patients may be early or late for scheduled FU visits can help clinicians and program planners to make informed decisions about the timing of FU visits that may be needed for different patient populations.

Interestingly, the finding that the majority of patients in this study did not demonstrate consistent patterns around the timing of follow-up visits has important implications for the way in which patient LTFU risk is assessed. Future research may benefit from focusing on the ways in which patient characteristics at treatment initiation can interact with visit- level factors in terms of influencing the proportion of patients who return for FU visits and when. The following discussion section outlines key findings and recommendations from the present study and is organized using the Andersen-Newman Framework of Healthcare Utilization [39].

### Predisposing Characteristics

While the majority of patients in our study had two or more follow-up visits after their initial baseline visit, 8% of patients had only a single follow-up visit. Predisposing characteristics at baseline were particularly relevant for both patterns of follow-up as well as time to first late or potential LTFU visit. Significantly more men had only one follow-up visit. While some evidence suggests that women are more likely to face barriers accessing ART [40], [41], in many settings, women are more likely to initiate ART [41], [42]. This is consistent with the findings in this study. Furthermore, some have suggested that men are more likely to initiate ART at a later WHO stage which places them at an increased risk of poor health outcomes including becoming LTFU or death [20], [42]–[44]. In our study, males also had a shorter median time to a first late visit as well as shorter time to missing a visit by ≥60 and ≥90 days. For women, the desire to be able to take care of their families and see their children grow up may be a strong motivator for adherence [45]–[48]. This can partly explain why married individuals in this study had more follow-up visits and were generally on ART for longer before experiencing a late visit compared to their non-married counterparts. While various aspects of social support can aid adherence through emotional backing, financial support, and by acting as direct reminders to take ART and attend follow-up visits [46], there is conflicting evidence with respect to the role of marital status [29], [44]. Disclosure of HIV status to one's marital partner can result in much needed social support [49] but can also lead to stigmatization, discrimination, and abandonment [50]–[54]. Interestingly, religious affiliations varied significantly with the number of follow-up visits. Religious activities [22], [23], [53] and ‘the belief that prayer, and not ART will heal’ was identified as a key reason why patients on ART may become lost to follow-up in Zomba and elsewhere [55]. These initial findings indicate the need to further study the potential complexities individuals face while negotiating ART, paying particular attention to the social conditions that exist at the time of initiation.

### Enabling Resources

Particular contributing factors matter more in the earlier versus later stages of ART treatment [12], [13], [21], [23], [25], [53], [55]. Given that we did not limit our analyses to patients who had been on treatment for a minimum duration (e.g., at least 1 year), not all included patients had the same opportunity to return for more FU visits. At the same time, by including patients who had been on ART for short periods of time in our study, we were able to explore the timing of FU visits that can occur earlier in the course of treatment. In our study, patients with only one follow-up visit had been on ART for a shorter period of time compared to patients with 2 or more follow-up visits, suggesting that the risk of becoming LTFU is higher in the earlier stages of ART. However, it is worth noting that a shorter time on ART has been associated with death in patients who become LTFU [43], [56]. Individuals who miss visits in the first year of treatment have reported to have higher risk of mortality [10], [18], [19]. At the same time, patients who have been on ART for longer periods of time often experience an improvement in health. For some, this improvement may lead them to believe that treatment is no longer necessary [12]. Feeling like ART is a burden and/or wanting to return to a ‘normal’ life [54] have previously been associated with being late for and missing scheduled follow-up visits, particularly for individuals who have been on ART for longer periods of time [57]. In the present study, median time on ART was longest in patients at *Late* visits compared to those at *Early* and *On Time* visits. Importantly, these findings suggest that the visit type in itself (e.g., *Late* vs. *Early*) can be used to indicate whether a patient will become LTFU at a later date and furthermore for what reasons. This has implications for patient care as the study findings can inform the development of tailored responses/strategies that are relevant at different stages in the course of ART treatment.

Enabling healthcare resources examined in the present study included whether a patient transferred into DI supported ART sites, the location of ART management, and whether they had ever been traced. While patients who transferred had a longer time before being late although a higher proportion of patients with only 1 FU visit had transferred in compared to patients with 2 or more visits. Transferring care from one location to another can be disruptive to care [58] and patients can become LTFU if the details of their transfer are not captured or recorded accurately. Enhanced documentation and stronger systems strengthening coordination between clinic locations is required as mature national programmes operationalize decentralization of care from referral centers to primary health care facilities.

Related to this is the specific location of ART management. Differences in follow-up across management locations (i.e., centralized vs. decentralized locations) were demonstrated in this study. In Malawi, a lower proportion of patients in decentralized care become LTFU [12], [21], [58]–[61]. This has been largely attributed to the fact that decentralized clinics in rural areas tend to be closer to patients homes requiring them to travel less, reducing transport costs [58], [59], [61]–[63]. The closer proximity can also make tracing more feasible from decentralized clinics. However, receiving care close to where an individual lives increases the likelihood that they will be recognized by others from their community. A fear of stigmatization and isolation may lead some patients to delay their return for a follow-up visit and/or choose to seek care elsewhere (e.g., an alternative decentralized clinic that is not as close to their primary residence) without being properly transferred [53], [61], [64], [65]. Indeed, patients in decentralized care were generally on ART for a shorter period of time before experiencing a late visit and *Late* visits had the highest proportion of patients in decentralized care. This suggests that even when care is close to the patient and potential transport costs reduced, patients can still face numerous challenges and complexities when negotiating routine follow-up. Patient volume, in this study categorized by the number of outpatient visits per year, varied across groups. The type and number of trained healthcare workers matters in the context of patient attrition [7], [57], [61], [66]–[68], although further study should explore the role of other healthcare/health-centre-specific factors [44].

The finding that there were some patients who were *Always On Time* that had been traced highlights potential opportunities for improvement in targeting of tracing. Patients who recently initiated ART and/or those with poor clinical status represent an important group for prioritization [69].

### Need Factors

Many clinical need factors demonstrated statistical significance across visit groups. While risk of becoming LTFU has been attributed to a poor clinical status at ART initiation [12], [14], [17], [18], [20], [23], [25]–[28], findings from the present study indicate that clinical status matters both in terms of whether a patient returns for more than one FU visit, as well as if they return *Early, On Time*, or *Late*. WHO stage at initiation was also associated with the number of follow-up visits with a higher proportion of patients in the only one follow-up visit group who initiated ART at WHO stage 4. Importantly, a late WHO stage at initiation has been associated with an increased risk of attrition and death [12], [18], [25], [56], [70]. Importantly, in the present study, patients who initiated ART at WHO stage 3 or 4 experienced a shorter time on ART before being late. This was true for all patients as well as those who had at least 2 FU visits.

Side effects due to ART have been previously associated with risk of becoming LTFU, particularly in the earlier stages of ART [12], [21], [26], [27]. The use of stavudine-based first line regimens in Malawi at the time of the study may have influenced the prevalence of side effects although access to second-line drugs remains limited [21], [71], [72]. While there were differences in proportions across the types reported, the highest proportion of side effects were noted in *Early* visits. This is a promising finding which suggests that patients are accessing care when they are acutely ill versus waiting for their scheduled FU visit. Our findings suggest that individuals who experience less severe or more manageable side effects (e.g., a rash) may delay their return to care. At the same time, our findings suggest that patients who are experiencing side effects do return, often a few days before expected. Moving forward, by identifying the type of side effects that impact on patterns of follow-up will help clinicians and front line staff to systematically determine which patients need to be followed more closely, to ensure they return for their next visit.

Adherence to ART was lowest in *Early* visits and upon return, the ART regimen was more likely to be held rather than continued or substituted. While among *Late* visits, the highest proportion of non-adherence was reported among patients who were ≥60 days late, a higher proportion was anticipated given that patients, theoretically, should have run out of pills within one week of missing their scheduled visit. In some cases, patients are likely getting pills from other sources (e.g., private sector, informal drug vendors, relatives) [73] or may even be under-dosing or sharing their medication with family members (e.g., due to stock-outs, confusion). Although patients who have been on treatment for longer periods may have stocked up on extra pills obtained over time [34]. Further investigation is needed to systematically identify these outside (of the clinic) sources of antiretrovirals as well as the frequency individuals are using them. Patients who have not only missed their visit but who also have truly run out of antiretrovirals should be distinguished from those who missed their scheduled visit but still have access to treatment as needed.

### Limitations

A large limitation of this and other studies of ART outcomes in resource-limited settings is the inability to link with death registries and determine the proportion of LTFU patients who are unascertained deaths. This is particularly relevant in the present analyses for patients who had advanced disease at ART initiation who had only 1 FU. Early mortality in patients starting on ART with profound immunosuppression and occult opportunistic infections unmasked by immune reconstitution can contribute to early LTFU [74]. Indeed, high rates of death particularly among patients in the earlier stages of treatment may result in overestimations of survival [75] and LTFU can underestimate true mortality [57]. Missing data is an important challenge with operational datasets derived from large clinical populations, particularly those in resource-poor settings. This can be attributable in large part to the lack of sufficient interest or resources needed to accurately collect, manage, and clean datasets [76]. Within DI, there are potential data consistency and validity issues specifically related to the way patient data is reported and recorded over time. In this study, inconsistencies occurred both within patients (i.e., visit to visit) and across patients. For example, weight can be recorded in one patient in kilograms and another in pounds, without clear indication of the units. Furthermore, missing data can be differential for some factors explored (i.e., weight may not be measured at follow-up if patient looks very sick). In the present study, missing data was also a concern particularly for key variables known to be relevant for LTFU. Related to this is our difficulty in identifying unascertained transfers out of the program. With the exception of having at least 1 FU visit, we chose not to restrict our analyses further to only patients with a minimum duration of FU; thus not all patients in our study had the same opportunity to return to the clinic for FU visits. However, through our approach, we were able to explore the risk of becoming lost in the early stages of treatment (e.g., early leavers). As well, the use of an operations and implementation research approach was appropriate here as we wanted to acknowledge and work with the real world conditions in which clinicians and front-line staff face daily (i.e., seeing a range of patients who show up at different expected times for their FU visits versus just those meeting specific eligibility criteria). Data used in the present study was extracted from routine monitoring and evaluation indicators and not through direct interviews with patients, limiting the variables available for exploration. Certain factors relevant for this study were neither accessed nor available including data about travel distance and transport costs. The rationale used to classify visits as early, on time, or late in this study may not be generalizable to other programmes and/or settings. Patients may have stocked up on ART over time which may be the case for patients who have been on ART for longer periods of time. As a result, patients may feel that it is unnecessary for them to attend a visit on time given that they are not in need of ART.

As clinicians and front line health workers are faced with many different types of patients, this study has strength in that both patient- and visit- level factors were explored and that patients with various FU times were included. The majority of patients did not demonstrate consistent patterns of follow-up over time; instead the likelihood of being on time, late or early varied across visits. These findings indicate that patients can miss scheduled visits and become LTFU at different stages in the course of their treatment for different reasons. They point to the dynamic complexities individuals may face over time, and as a result the need for a variety of responses that address the ongoing and changing challenges to improving patient retention overall.
